# A Soft Sensor for Measuring the Wear of an Induction Motor Bearing by the Park’s Vector Components of Current and Voltage

**DOI:** 10.3390/s21237900

**Published:** 2021-11-26

**Authors:** Natalia Koteleva, Nikolay Korolev, Yuriy Zhukovskiy, Georgii Baranov

**Affiliations:** 1 Educational Research Center for Digital Technologies, Saint Petersburg Mining University, 191106 Saint Petersburg, Russia; korolev_na@pers.spmi.ru (N.K.); zhukovskiy_yul@pers.spmi.ru (Y.Z.); 2 Department of Robotics and Industrial Systems Automation, Saint Petersburg Electrotechnical University “LETI”, Professora Popova 5, 197376 Saint Petersburg, Russia; gdbaranov@etu.ru

**Keywords:** soft sensor, Park’s vector, induction motor bearing, ANN-classifier

## Abstract

This paper presents a methodology for creating a soft sensor for predicting the bearing wear of electrical machines. The technique is based on a combination of Park vector methods and a classifier based on an artificial neural network (ANN-classifier). Experiments are carried out in laboratory conditions on an asynchronous motor of AIR132M4 brand. For the experiment, the inner rings of the bearing are artificially degraded. The filtered and processed data obtained from the installation are passed through the ANN-classifier. A method of providing the data into the classifier is shown. The result is a convergence of 99% and an accuracy of 98% on the test data.

## 1. Introduction

Today’s trend in industrial companies is to improve automation systems in all areas of operation [1,2]. This is primarily due to the digitalization of production [3]. The power supply industry is no exception. Automated electric drives are getting to become a major part of any industry, and their efficiency defines the growth and development strategy for companies [4,5]. The digital transformation of the energy sector is rapidly bringing new solutions to the market [6,7]. This development goes hand in hand with an uncontrolled increase in computing and instrumentation capacity. Current sensors and measurement principles for key process parameters are lagging far behind. However, sensor developments are much slower than necessary to keep up with the trend towards digitalization. The electricity supply area is no exception. There are a number of parameters that have to be measured, but for a number of reasons that is still not possible. The same trend can be seen in monitoring and maintenance systems for electrical machines. This in turn prevents the development of evaluation maps drive conditions for different process equipment topologies [8,9].

The induction motor is the key link and the most susceptible to wear and tear. An analysis of company data [10,11,12] identifies a segment of frequently occurring faults and research related to them, of which bearing defects are to be highlighted. Bearing failure leads to deviations in the mating components due to the multi-component nature of electrical machines. Untimely detection leads to accelerated wear and gradual degradation of coupled mechanical (couplings, reducers, etc.) and electrical (stator and rotor windings) parts of machines. It is of scientific interest to search for software and hardware solutions ensuring detection of bearing faults at the minimum stages of development with a minimum number of sensors.

In this context, the development of a soft sensor is an urgent task. It enables real-time measurements of various parameters that can subsequently be used to diagnose and assess the technical condition of electrical machines. Currently, the development of sensors and special methods of diagnostics and technical condition monitoring is carried out separately in science. 

The paper (Ewert, P. et al.) [13] focuses on a system for the online monitoring of IM bearings and a subsequent fault diagnosis based on analysis of vibration measurement data. The bearing condition evaluation is performed by an appropriately trained neural network (NN) based on spectral analysis and mechanical vibration envelope analysis. In the absence of physical access or pre-built vibration sensors, the method is difficult to apply compared to the current analysis.

Experimental results from researchers (Lo, N.G. et al.) [14] demonstrate the effectiveness of discrete wavelet transforms in combination with Clarke shape indicators for classifying bearing and gear faults. The discrete wavelet transform is used as a filtering method to extract different frequency bands. The indicators used in this work are geometric shape descriptors derived from the Clarke transform. These indicators give a particular shape in the presence of a mechanical fault. The results of these experiments demonstrate the effectiveness of the discrete wavelet transform for the classification of bearing faults.

The article (Sunder, M. et al.) [15] presents an approach to bearing fault detection using Park’s vector. This study aims to analyze the deformation of the hodograph with respect to the reference one in the presence of bearing defects. The researchers have performed numerical analyses at varying degrees of defect on a large number of machines, but do not address the issue of software defect recognition.

The paper (Elbouchikhi, Elhoussin, et al.) [16] focuses on rolling bearing fault detection in induction machines based on stator current analysis. In particular, it is proposed to treat stator currents using the Hilbert–Huang transform. This approach is based on two steps: empirical mode decomposition and the Hilbert transform. The proposed approach is applied to detect bearing faults in asynchronous machines with several fault degrees.

The authors in the paper (Kompella, K.D. et al.) [17] describe discrete wavelet transform (DWT), stationary wavelet transform (SWT) and wavelet packet decomposition (WPD). In addition, a comparative analysis is performed using different fault identification parameters. The complexity of this method lies in the processing and separation of the diagnostic data criteria. 

The work by (Fournier, E. et al.) [18] discusses a method of developing a reference signal for a fault-free system. Indications of an appropriate fault condition are identified by comparing the actual signal with the reference signal. The described method, compared with others, is most suitable for a large power segment of asynchronous motors considering the load and actuator variants. 

Authors (Gyftakis, K.N. et al.) [19] present a new Filtered Park/Filtered Extended Park (FPVA/FEPVA) Vector Approach for broken rotor bars, which allows for the adjusting of machine design features. The other most common defects and the machine’s use of diagnostic data processing are of interest.

There have been many studies on the diagnosis of induction motors using the Park vector [20,21,22], but little attention has been paid to the treatment of vector trajectories. 

There are a number of papers that show the advantage of using a soft sensor and the principles of its development for different industries [23]. For example, the article [24] deals with one of the issues of failsafe electric drive with direct field-oriented control of induction motor (Direct Field Oriented Control). An important point is the incorrect operation of the control system in case of a faulty Hall-effect current sensor. The use of a neural network to detect the status of the stator current sensor was suggested. The work convolutional neural network (CNN) fusing a frequency domain feature matching algorithm (FDFM) is used for the diagnosis of rolling bearings [25].

By analyzing available approaches, the authors propose to combine electromechanical machine diagnostics, Park theory and neural network theory. The next step is to create a software sensor that can be used to diagnose the technical condition of electromechanical equipment. 

The main hypothesis of the paper is that a soft sensor, which is a mathematical apparatus combining the Park vector transformation and a classifier based on an artificial neural network (ANN-classifier), will enable real-time (or a frequency suitable for use in control systems) detection of bearing defects in electromechanical machines. The bearing is chosen as the main diagnostic unit in this case because the bearing is more subject to wear due to frictional forces than other parts of the machine, as well as its successive failure of all other parts.

## 2. Materials and Methods

The basic idea of the paper is the Park (Gorev) vector transformations [26], consisting of the transition to a two-phase current system (*i_d_, i_q_*) in the rotating coordinate system-dq from the three-phase current system consumed by the induction motor (*i_A_, i_B_, i_C_*,) [27] by the following mathematical Equations (1) and (2). These equations are valid for the real electric motor [28,29,30].

(1) $$i_{d}=\left[\sqrt{\frac{2}{3}}\right]\times i_{A}-\left[\sqrt{\frac{1}{6}}\right]\times i_{B}-\left[\sqrt{\frac{1}{6}}\right]\times i_{C}$$

(2) $$i_{q}=\left[\sqrt{\frac{1}{2}}\right]\times i_{B}-\left[\sqrt{\frac{1}{2}}\right]\times i_{C}$$

where

*i_d_, i_q_*—currents consumed by an asynchronous motor (AM) in a 2-phase rotating coordinate system-dq;

*i_A_*, *i_B_, i_C_*—currents consumed by an asynchronous motor (AM) in a 3-phase rotating coordinate system-ABC.

Assuming that the asynchronous motor is a reference and is a three-phase symmetrical active-inductive load, the following equations are valid (3) and (4)

(3) $$i_{d}=\left[\frac{\sqrt{6}}{2}\right]\times i_{max}\times \sin\left(\omega t\right)$$

(4) $$i_{d}=\left[\frac{\sqrt{6}}{2}\right]\times i_{max}\times \sin\left(\omega t-\frac{\pi }{2}\right)$$

where

*i_max_*—maximum amplitude value of phase current, A;

*ω*—power angular frequency, rad/s;

*t*—time, s.

In a coordinate system-dq, the generalized vector of *I_S_* current is written as (5) and is valid for (1–4).

(5) $$I_{S}=i_{d}+j\times i_{q}$$

**Figure 1:**
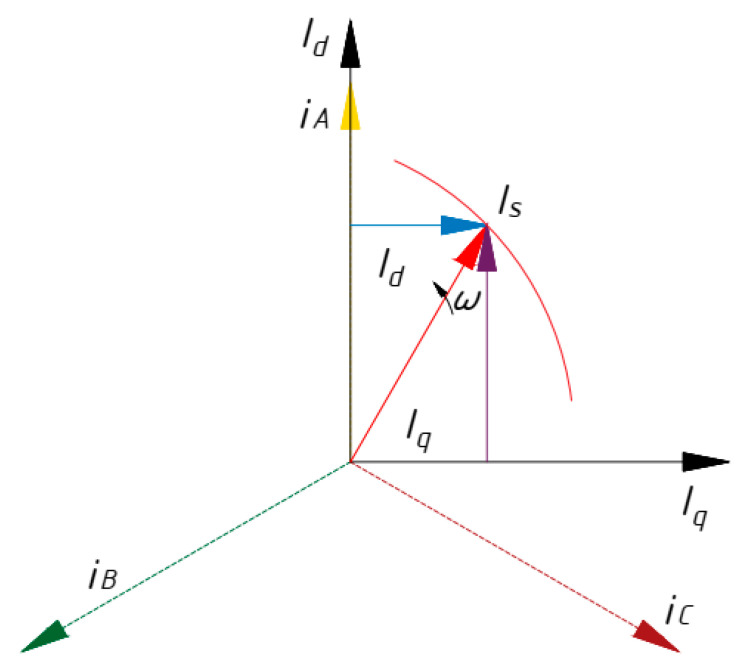
Vector diagram of Park’s transformation (Gorev).

These mathematical transformations are shown in a vector diagram, Figure 1.

According to Equation (5), the generalized vector in the complex plane will describe a trajectory—the hodograph. The distortion of the real current vector trajectory with respect to the reference one is observed.

If the asynchronous motor is defective in terms of stator, rotor or mechanical damage, the hodograph of the generalized current vector changes relative to the reference. If one of the stator phases is damaged, the hodograph degenerates into an ellipse. The proportional change in ellipticity and width of the Park’s vector hodograph corresponds to the defect level [21]. Disturbance in the rotor causes the hodograph to degenerate into a complex shape [22]. Damage to the mechanical part of the motor gives rise to circular distortions and variations in the width of the described hodograph [20]. The study of complex trajectories makes it possible to determine the types and intensity of the influence of defects on the rotor speed and torque on the motor shaft in a comprehensive manner.

In this paper, a neural network classifier is used to identify changes in the Park vector, which allows for estimating changes in the state of the Park vector and signals the onset of a defect in a timely manner.

**Figure 2:**
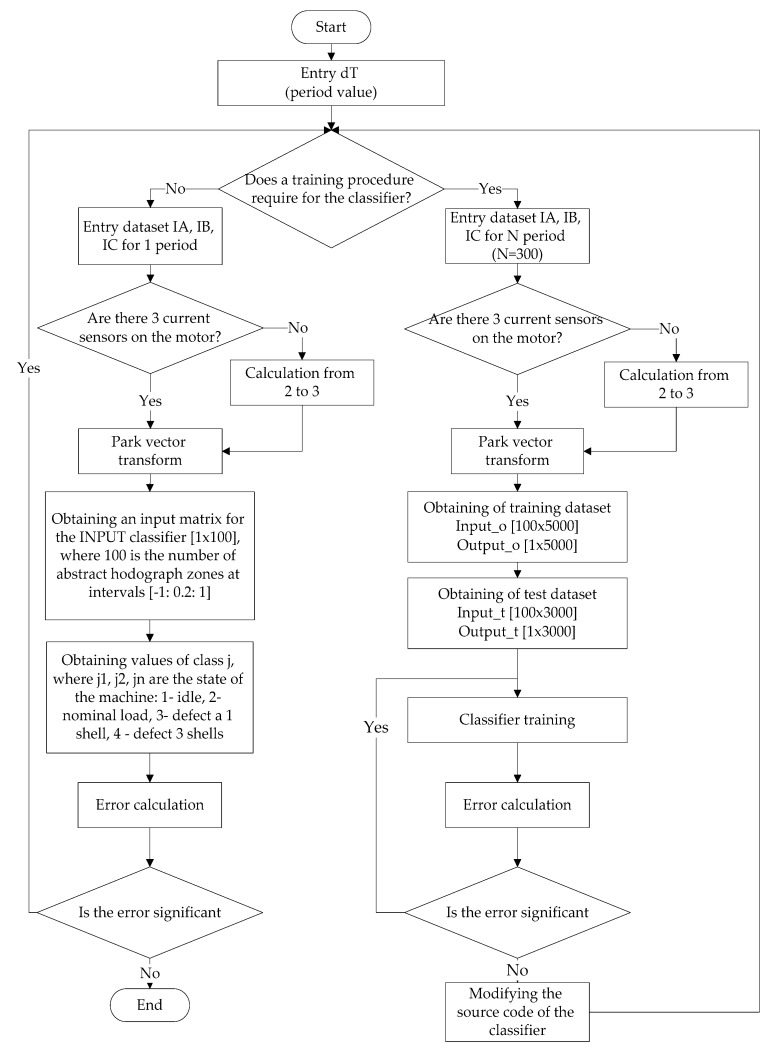
The algorithm for ANN classifier.

However, in order to use such a classifier, it needs to be adapted to process the data coming in the real-time mode [31,32]. Figure 2 shows the algorithm used in developing the ANN-classifier [33].

A brief list of actions, according to this algorithm, is as follows: supply values equal to one given time period to the input of the model; obtain values for the Park vector; generate a matrix of values for the input to the classifier; obtain the result. The basic idea, in this case, is the idea of the special formation of the input vector for the ANN-classifier. As such a vector is taken as a vector showing how many times the data vector falls into one or another abstraction region of the Park vectors’ hodograph. In total, the hodograph was divided into 100 abstraction zones from minus 1 to 1 with a step of 0.2 (10 segments on the imaginary and real axis). Thus an array of size (100 × 1) is fed to the input of the ANN-classifier, where the value of each element of the array is the number of points falling into a given abstract zone.

In order to train and test the neural network, the data captured during the experiment were divided into groups. A total of 600,000 values were captured during the experiment. Ten percent was used for training. The rest of the data were used for testing. The important point in training and testing the data was to keep the right sequence. For the network to work correctly, an unbroken vector of data was required for the 1st period of operation. The data were therefore pre-divided into these periods with certain labels.

## 3. Experiments

**Figure 3:**
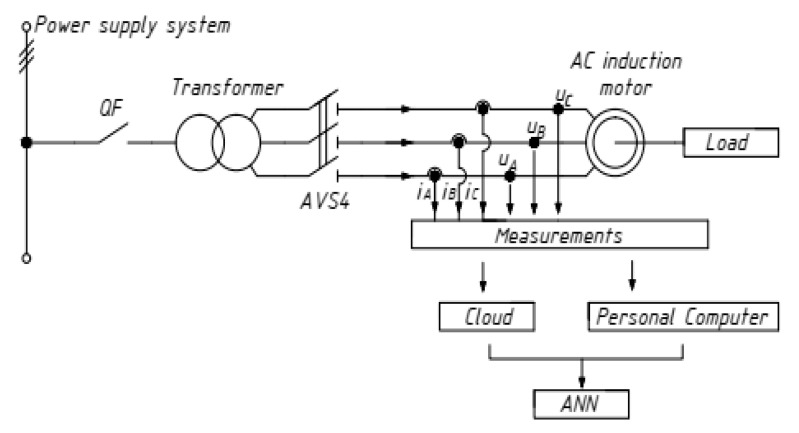
Asynchronous motor AIR132M4, wiring and measurement diagram.

The object of the research is an asynchronous motor with the parameters given in Table 1. It is powered from a 50 Hz mains supply in a continuous duty mode S1 and a constant shaft load. The appearance and wiring diagram of the AM is shown in Figure 3. A similar motor in generator mode gives a constant load.

**Figure 4:**
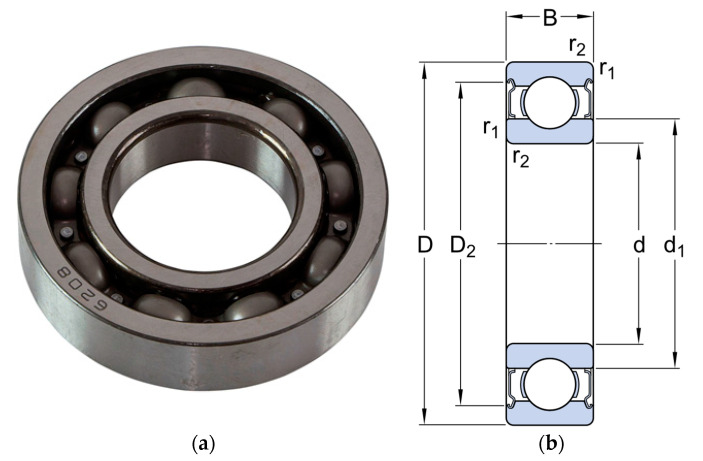
Ball bearings of series—6208: (**a**)—general view; (**b**)—sketch of product size.

The impact of bearing failure has the most intense distortion of the magnetic field in the air gap, as shown in Figure 4, as well as in [34]. This type of defect has an increased rate of development, resulting in rapid degradation of the mechanical and associated electrical parts of the motor [35].

According to the motor datasheet, the motor AIR132M4 is fitted with deep groove ball bearings series-6208 with the following technical data (Figure 4 and Table 2).

According to Figure 4, bearing 6208 has the following dimensions, Table 2.

**Figure 5:**
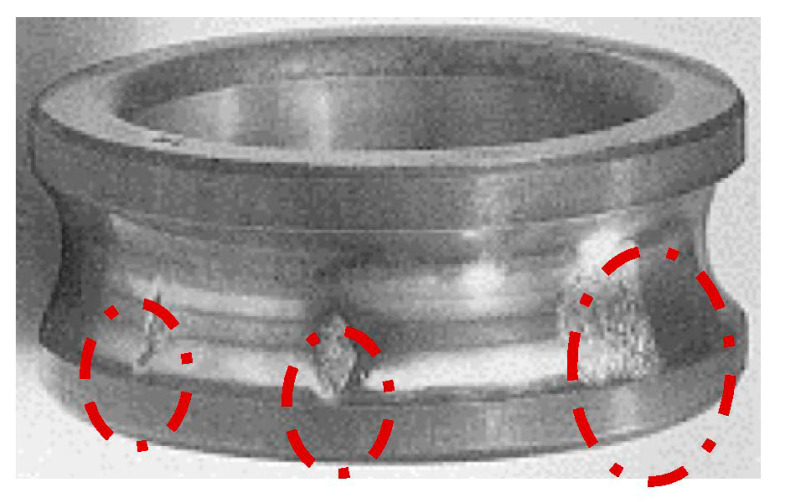
Degradation shells of a deep-groove ball bearing.

The procedure for the experiment is to artificially degrade the inner rings of the bearing as a number of friction-induced degradation shells in the deep-groove ball bearing. The degradation shells are shown in Figure 5.

The experiment was carried out on four states of the machine:

1. Reference motor operation without load at idle speed
2. Motor operation in reference condition at rated load
3. Motor operation with one shell in the inner ring of the bearing
4. Motor operation with three shells in the inner bearing ring

Tests in cases 3 and 4 were carried out at nominal load.

## 4. Results & Discussion

**Figure 6:**
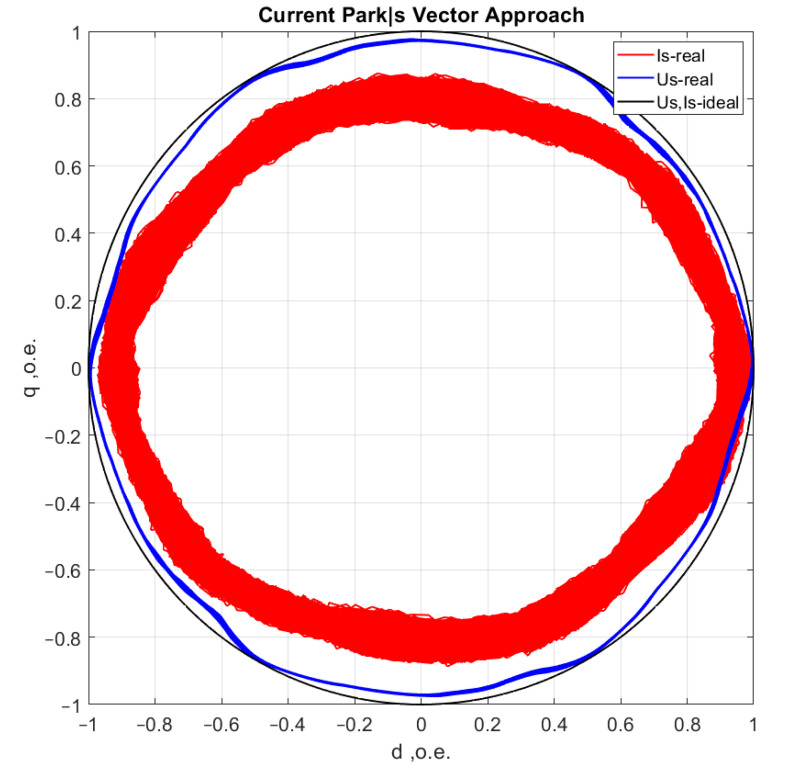
Reference motor operation without load at idle speed.

**Figure 7:**
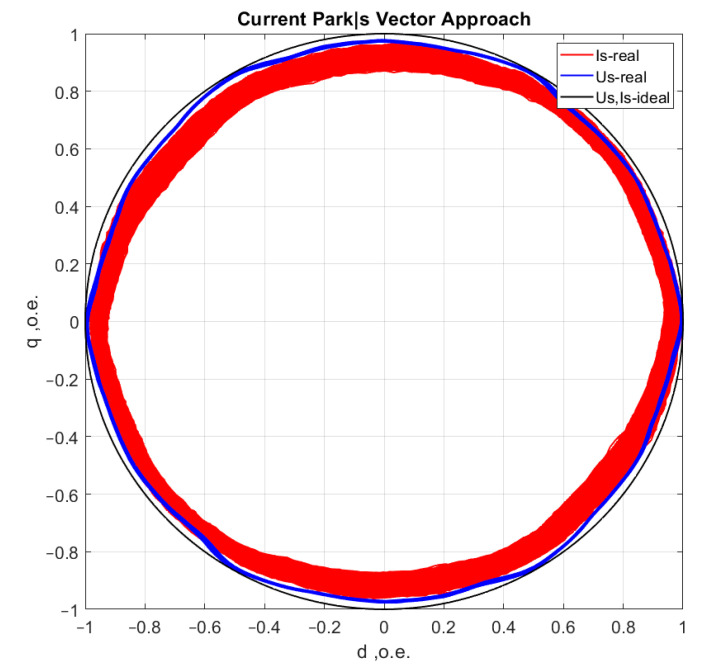
Motor operation in reference condition at rated load.

**Figure 8:**
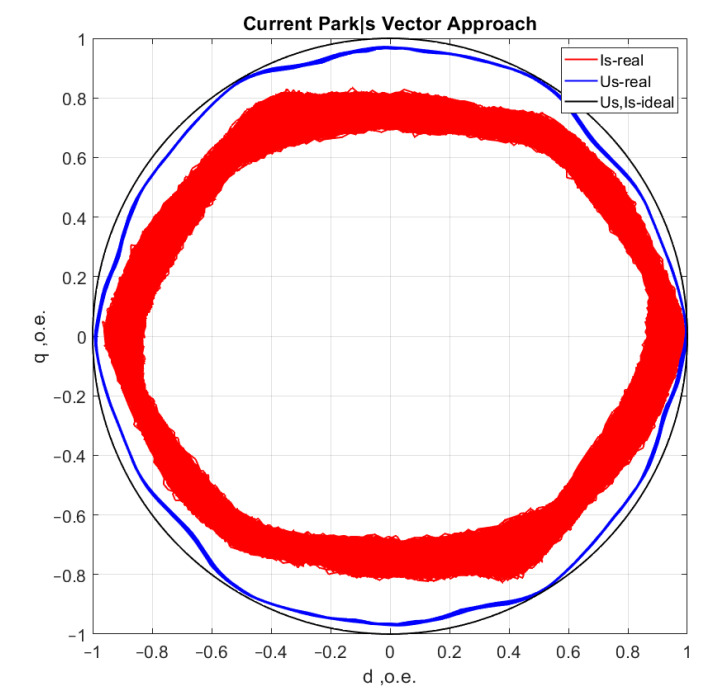
Motor operation with one shell in the inner ring of the bearing.

**Figure 9:**
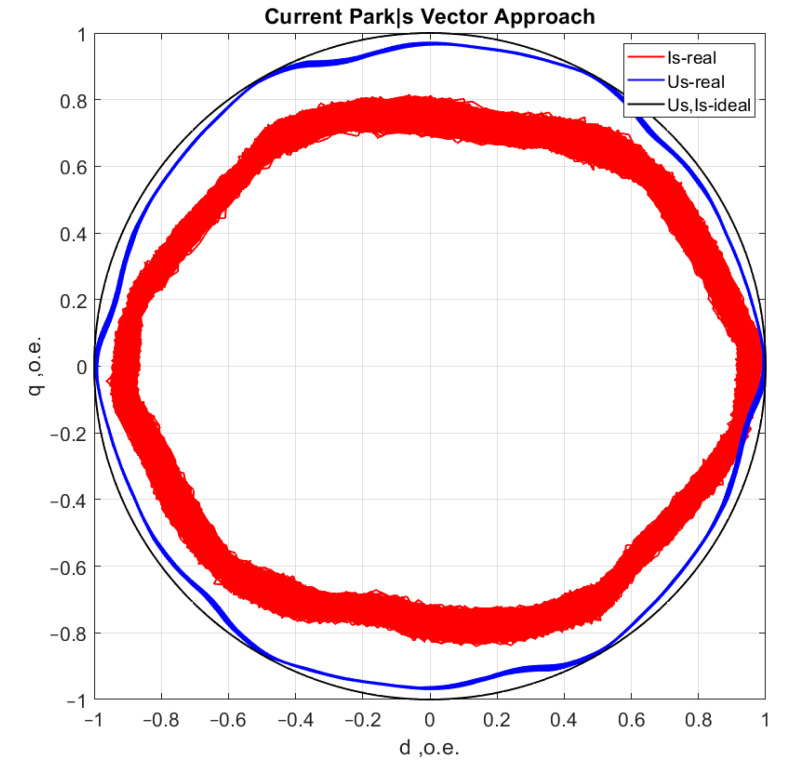
Motor operation with three shells in the inner bearing ring.

Figure 6, Figure 7, Figure 8 and Figure 9 show the data obtained during the experiment.

A visual assessment of the hodographs in Figure 6, Figure 7, Figure 8 and Figure 9 makes it possible to assert that the Park vector changes its state depending on the cases under consideration. The additional evidence for the expediency of using Park’s vector is shown below.

Study [30] on the spectral analysis of the current consumption of an electric motor highlights the characteristic frequencies by which the presence of a defect can be determined (6). The initial stage of bearing wear is the occurrence of contact parts with rolling elements, namely balls, on the inner or outer ring.

(6) $$f_{rb}=\frac{n}{2}\times f_{rm}\times \left[1\pm \left(\frac{D_{ball}}{D_{pit}}\times cos\;\beta \right)\right]$$

where

*f_rm_*—rotor speed;

*n*—number of balls in the bearing;

*β*—contact angle;

*D_pit_*—diameter of the circumference of the ball centres;

*D_ball_*—ball diameter.

**Figure 10:**
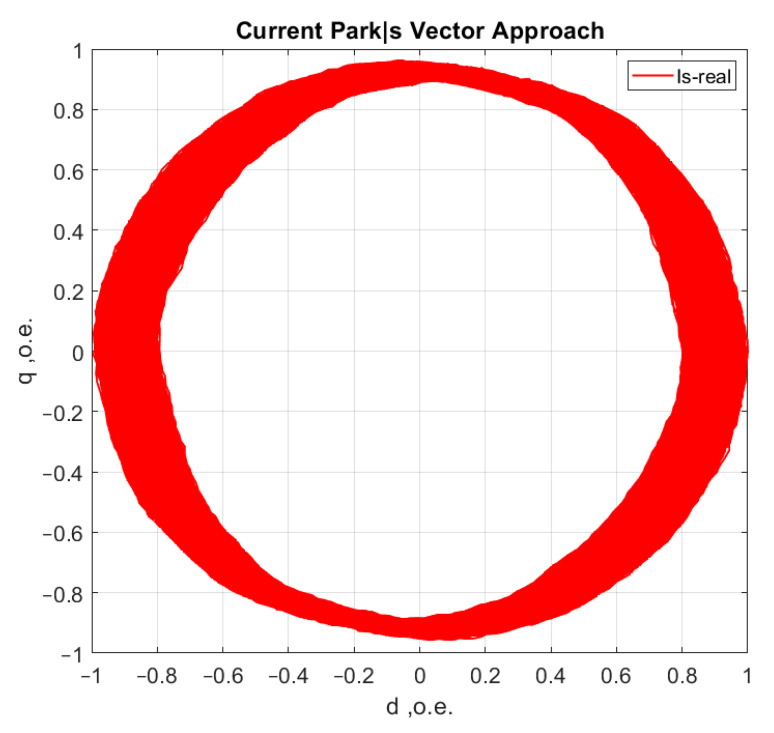
The hodograph at the start of the defect.

**Figure 11:**
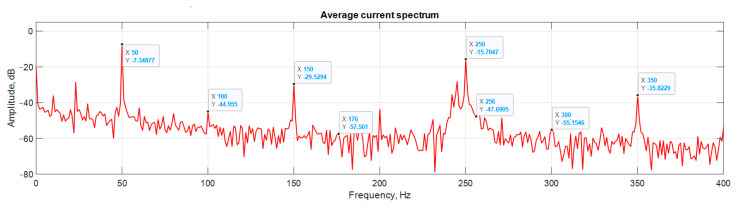
Spectrum at the start of the defect.

**Figure 12:**
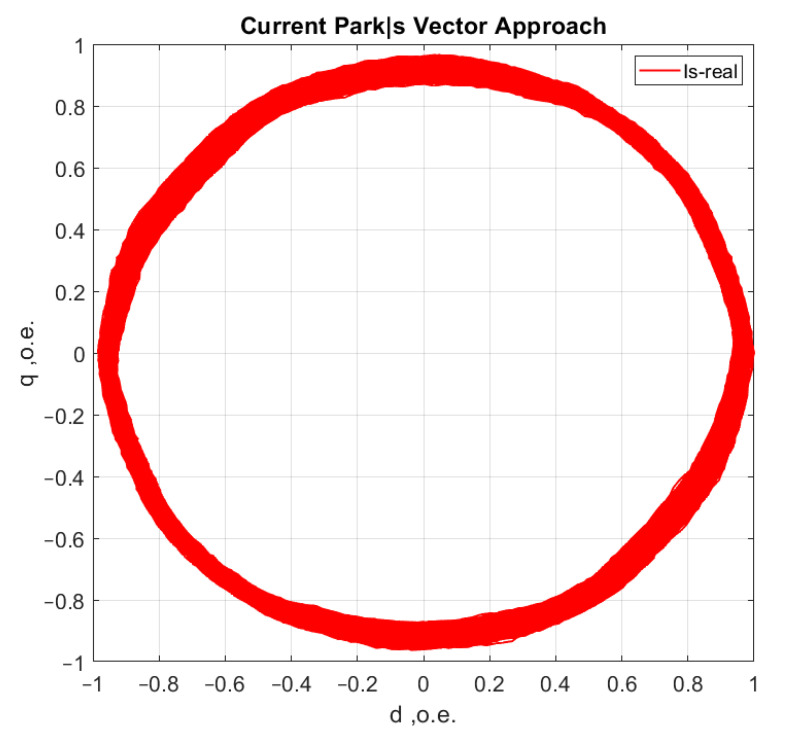
The hodograph without any defects.

**Figure 13:**
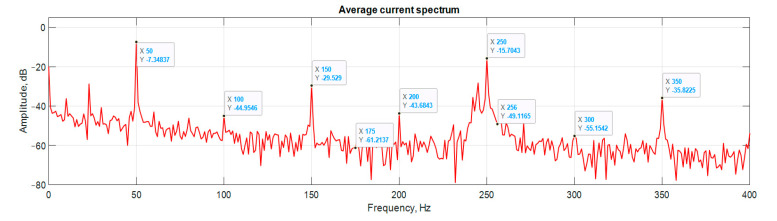
Spectrum without any defects.

**Figure 14:**
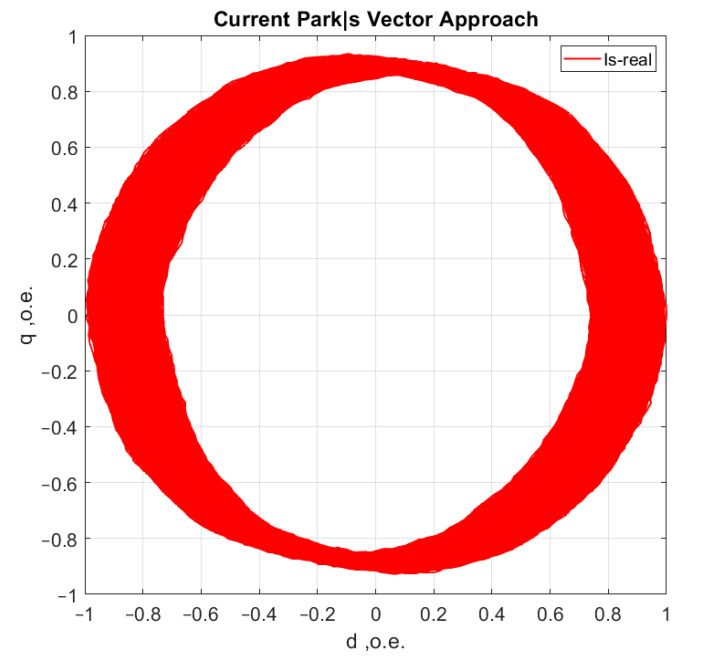
The hodograph at medium defect.

**Figure 15:**
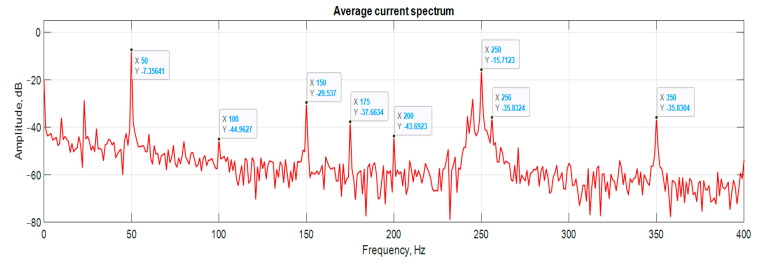
The spectrum at medium defect.

Compared to spectrum analysis (FFT) [36], where part of the information is lost due to the use of low and high-pass filters and is in the noise region (−75–80 dB), it is almost impossible to detect the initial stages of the defect. The occurrence of a defect (in our case a bearing defect) in the hodograph can be detected earlier as a distortion in its trajectory (Figure 10), which the spectrum does not provide. The expected calculated bearing fault frequencies according to (6) and Table 2 at *cos β* = 1 are *f_rb_* = 175 Hz, 256 Hz. By analyzing the hodographs and spectra (Figure 10, Figure 11, Figure 12, Figure 13, Figure 14 and Figure 15) changes in the current hodograph (Figure 10) are observed as the defect degree changes. However, it is difficult to isolate the peaks of the frequency components of the spectrum, which is an advantage of the generalized current vector method. However, due to the visual identification of faults in the trajectory distortion hodograph, it is not possible to construct a fault-level estimation system.

**Figure 16:**
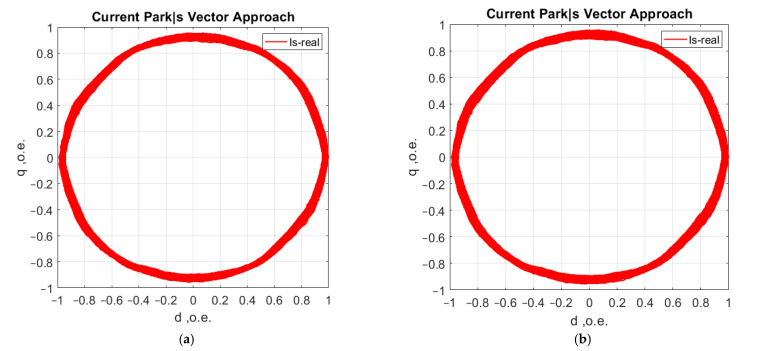
AM current consumption hodographs: (**a**) when recording A, B, C phase currents; (**b**) when recording A and C phase currents.

Further analysis of the method has highlighted a number of advantages. The first is that only two current sensors (Hall-effect compensated current sensors) are required to detect faults, as shown in Figure 16. The third phase current is determined indirectly [37,38,39].

**Figure 17:**
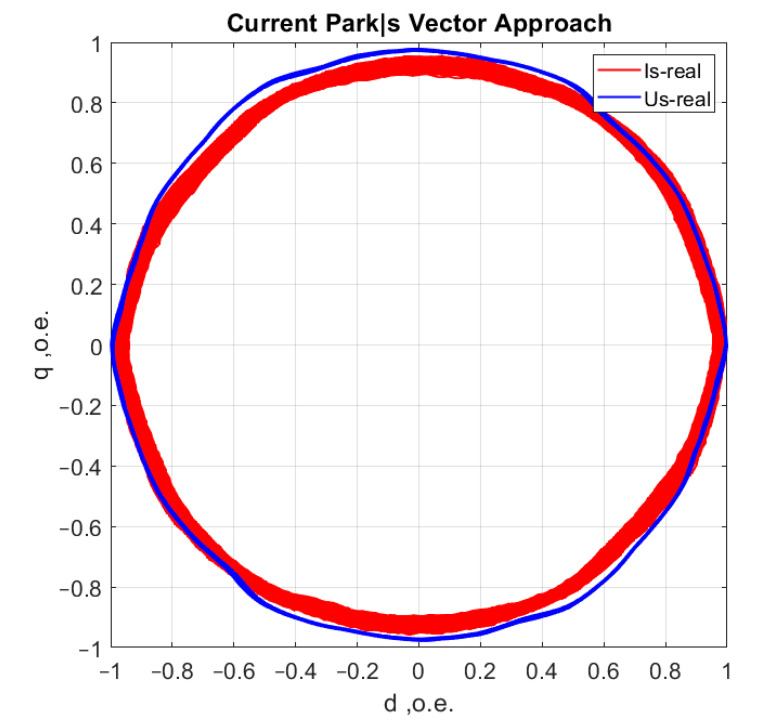
AM current consumption (red) and supply voltage (blue).

The second is achieved by a synchronous recording of the phase voltage currents (uA, uB, uC), which eliminates the influence of mains distortion. Distortions due to mains quality [40] have to be mutually excluded in the analysis from the asynchronous motor hodograph, (Figure 17).

The information value of the hodograph is quite high, but in addition to the trajectories already obtained and their changes to which certain stages and types of both individual and complex defects correspond [41].

**Figure 18:**
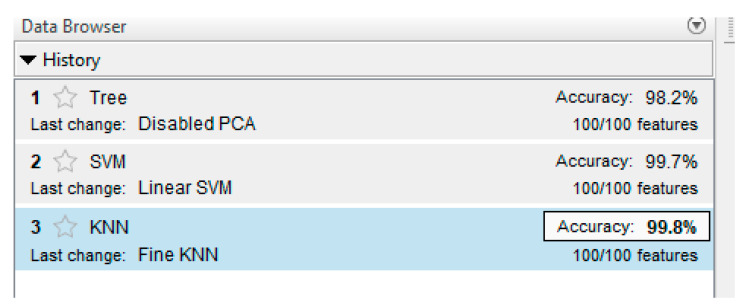
ANN-classifier learning result.

Figure 18 shows the results of training the ANN-classifier. The classifier was trained using three methods: Decision Tree [42], Support Vector Machine [43] and K-nearest neighbors [44].

**Figure 19:**
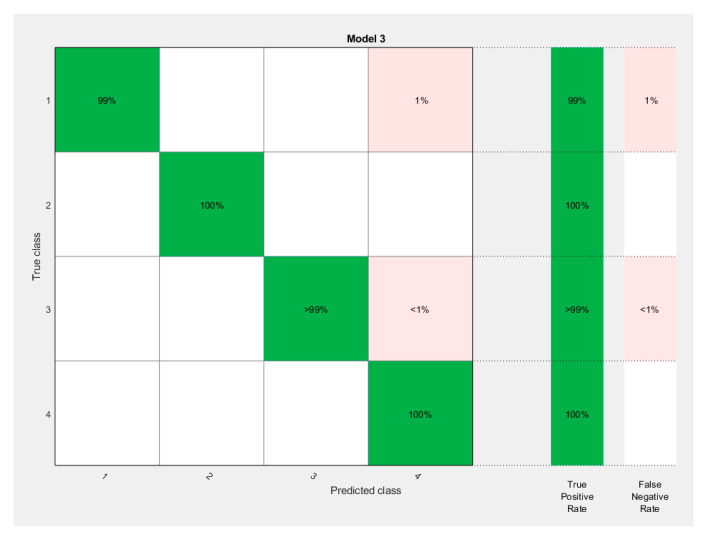
ANN-classifier learning result. Confusion matrix.

As can be seen in the Figure, the best was K-nearest neighbors, with an accuracy of 99.9%. This is a good result. Figure 19 shows the result as a confusion matrix.

Testing the ANN-classifier algorithm on real data gave significant results as well. The accuracy was 98%. The excessively high accuracy of the results is due to the idealized conditions of the experiment. Under real-life conditions, the machine can be subject to several types of defects [45]. It may operate under disturbing conditions and at different modes. This paper has shown the validity of the proposed methodology, however, for real objects it may not give such high results. However, the authors believe that in order to make it applicable to real objects it is necessary to break the Park hodograph into smaller abstract zones. This would solve the problem of possible overlapping distortions of Park’s vector.

## 5. Conclusions

In the course of the work, on the whole, it was possible to prove the consistency of the hypothesis. Machine defects can be estimated using a mathematical apparatus that allows the combination of Park’s vector transformation and an ANN-classifier. Using this mathematical apparatus in the real-time mode with a certain periodicity allows us to work out the fully-fledged soft sensor for measuring a certain type of defect. However, the accuracy of the results obtained in the work may be reduced in conditions of real operation of electrical machines. The authors emphasize that the basic idea of the method when these conditions occur will not change. Only the components of this methodology will change, for example, the number of abstract zones in the division of the Park hodograph will increase.

The authors thus offer a method of determining the condition of a bearing in near real-time, or with a minimum decision time. The authors have shown that it is possible to tell just by one spin of the Park hodograph, i.e., by one period (0.02 s) that a bearing has developed a defect. This can be carried out by applying a special ANN-classifier, which in turn determines which of the predefined abstract zones the current Park vector values fall into. The division into such zones or quadrants is the basic tool for improving the accuracy of the soft sensor. The closer the bearing conditions are to ideal, the larger the abstract zones that are allocated. However, it must be remembered that the allocation of such zones is initially laid down and their extension is not possible during operation, i.e., on the go. Therefore, experiments under real-field conditions should be carried out beforehand to determine the necessary width of the considered abstract zones for the Park hodograph.

## Figures and Tables

**Table 1:** Nameplate and calculation data for asynchronous motors.

Motor Brand	*P_nom_*, kW	Current, *I_nom_*, A	*n* r/min	*cos φ*	*η_m_*, %	*λ*	*K_p_*	*K_i_*	Parameters of Equivalent Circuit				
									*L_s_*, H	*L_r_*, H	*L_m_*, H	*R_s_*, O	*R_r_*, O
AIR132M4	11.00	23.40	1450	0.82	87.1	2.3	2.2	6.8	0.146	0.148	0.140	0.522	0.306

*P_nom_*—nominal motor power, kW; *I_nom_*—nominal motor current, A; *n*—motor shaft speed, r/min; *η_m_*—motor efficiency, o.e.; *cos φ*—power coefficient, o.e.; *λ*—overload capability, o.e.; *K_p_*—multiplicity of starting torque, o.e.; *K_i_*—starting current multiplicity, o.e.; *L_s_, L_r_*, *L_m_*, *R_s_*, *R_r_*—parameters of asynchronous motor equivalent circuit, H, O.

**Table 2:** Bearing specifications 6208.

Bearing	d, mm	D, mm	B, mm	d_1_, mm	D_2_ mm	r_1_, r_2_, mm
6208	40	80	18	52.6	69.8	min. 1.1

d—inner diameter, mm; D—outer diameter, mm; B—Width, mm; d_1_—Inner ring diameter, mm; D_2_—Outer ring diameter, mm; r_1_, r_2_—Outer ring chamfer size, mm.
